# PRDX6 controls multiple sclerosis by suppressing inflammation and blood brain barrier disruption

**DOI:** 10.18632/oncotarget.5205

**Published:** 2015-08-17

**Authors:** Hyung-Mun Yun, Kyung-Ran Park, Eun-Cheol Kim, Jin Tae Hong

**Affiliations:** ^1^ Department of Maxillofacial Tissue Regeneration, School of Dentistry and Research Center for Tooth and Periodontal Regeneration (MRC), Kyung Hee University, Dongdaemun-gu, Republic of Korea; ^2^ College of Pharmacy and Medical Research Center, Chungbuk National University, Cheongju, Chungbuk, Republic of Korea

**Keywords:** PRDX6, EAE, demyelination, astrocytes, inflammation, multiple sclerosis, Pathology Section

## Abstract

Multiple sclerosis (MS) is a complex disease with an unknown etiology and has no effective medications despite extensive research. Antioxidants suppress oxidative damages which are implicated in the pathogenesis of MS. In this study, we showed that the expression of an antioxidant protein peroxiredoxin 6 (PRDX6) is markedly increased in spinal cord of mice with experimental autoimmune encephalomyelitis (EAE) compared to other PRDXs. PRDX6 transgenic (Tg) mice displayed a significant decrease in clinical severity and attenuated demyelination in EAE compared to wide type mice. The increased PRDX6 expression in astrocytes of EAE mice and MS patients reduced MMP9 expression, fibrinogen leakage, chemokines, and free radical stress, leading to reduction in blood-brain-barrier (BBB) disruption, peripheral immune cell infiltration, and neuroinflammation. Together, these findings suggest that PRDX6 expression may represent a therapeutic way to restrict inflammation in the central nervous system and potentiate oligodendrocyte survival, and suggest a new molecule for neuroprotective therapies in MS.

## INTRODUCTION

Multiple sclerosis (MS) is a chronic inflammatory disorder of the central nervous system (CNS) [1]. Its disease is characterized by immune cell infiltration, demyelination, axonal damages, and a reactive astrogliosis [2-3]. Experimental autoimmune encephalomyelitis (EAE) by injection of a myelin antigen, which is activated by myelin-specific T cells is the most widely accepted animal model to study MS [4]. Although the disease process is understood very well, the critical factors inducing the etiopathogenesis of MS remain largely unknown.

Oxidative stress plays an important role in the pathogenesis of EAE and MS [1, 5]. Many studies have been showed increased free radical levels in MS patients, and oxidative stress has been implicated as mediators of demyelination and axonal damage in both EAE and MS [6–7]. Oxidative stress is controlled by antioxidant proteins. peroxiredoxins (PRDXs) are newly discovered family of antioxidant enzymes [8]. Uniquely, PRDX6 has a single conserved cysteine residue (1-Cys PRDX), utilizes glutathione (GSH) as the physiological reductant, and removes cellular reactive oxygen species (ROS) and protect cells from ROS-induced cell damage and death [9]. PRDX6 was only expressed in astrocytes of mouse brain among the six mammalian PRDXs (PRDX1 - 6) [10], and it also was reported to be elevated in reactive astrocytes of human PD patients and dementia patient brains [11], and implying unique roles of PRDX6 in astrocytes. However, the potential roles of PRDX6 on MS have not yet been studied.

In the present study, we investigated the role of PRDX6 using MS patient samples and EAE-generated mice. Our study provides that PRDX6 has pathophysiological functions in inflammatory progression of MS.

## RESULTS

### PRDX6 is expressed in mouse spinal cord and increased in EAE and MS lesions

**Figure 1:**
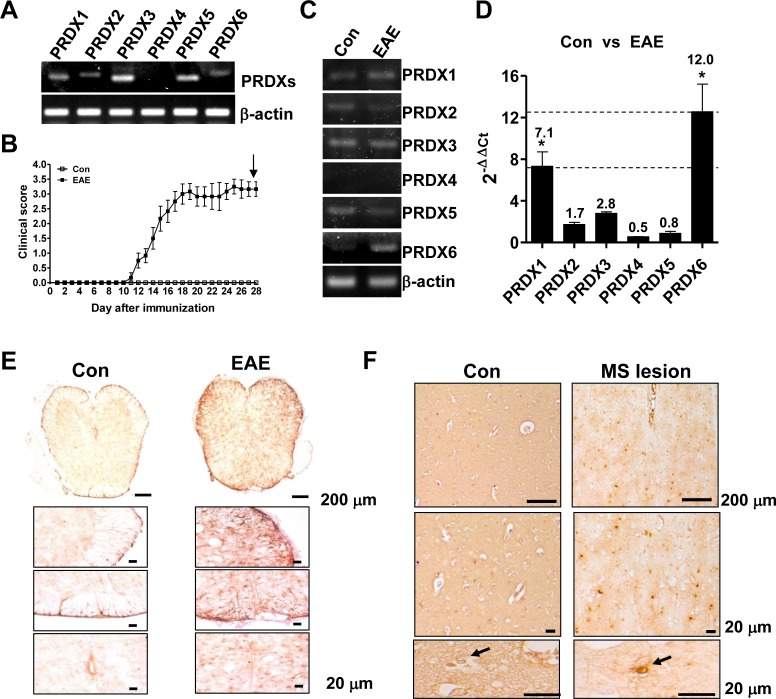
EAE lesions are characterized by enhanced PRDX6 **A.** mRNA level of PRDXs (PRDX1, PRDX2, PRDX3, PRDX4, PRDX5, and PRDX6) was detected in mouse spinal cord using RT-PCR. **B.** – **D.** C57BL/6 mice were sensitized to MOG_35-55_, as described in Materials and Methods **B.**. 28 days after EAE induction, relative mRNA level of PRDXs in spinal cord was compared with control spinal cord using RT-PCR **C.** and real-time qPCR **D.**. **E.**, **F.** Immunohistochemistry for PRDX6 is shown in EAE mouse spinal cord **E.** or human MS lesions **F.**. Representative stainings from three independent experiments are shown. All values are presented as the mean ± SEM. *Significant difference between control (con) and EAE (*P < 0.05).

In order to investigate a potential relevance of PRDXs in MS, we first examined the expression of PRDXs in spinal cord. Except for PRDX4, PRDXs were expressed in mouse spinal cord (Figure 1A). After EAE induction for 28 days (Figure 1B), DNA microarray and RT-PCR were carried out in the spinal cord of animals (Figure 1C, S1_Table, and S1_Dataset 1). As shown in Figure 1C, S1_Table, and S1_Dataset 1, the mice sensitized with MOG_35-55_ peptides showed a marked increase in PRDX6 mRNA level in the spinal cord compared with control mice. We quantitatively validated the drastically increased mRNA level of PRDX6 compared to other PRDXs using quantitative real-time polymerase chain reaction (PCR) (Figure 1D). It is also confirmed the expression of PRDX6 in the spinal cord (S1-Figure). PRDX6 was strongly expressed on cells with astrocyte-like morphology in both EAE (Figure 1E) and human MS lesions (Figure 1F). These data imply that the up-regulation of the antioxidant PRDX6 may be a critical role in the progression of MS.

### PRDX6 suppresses the clinical severity and neuropathology of EAE

**Figure 2:**
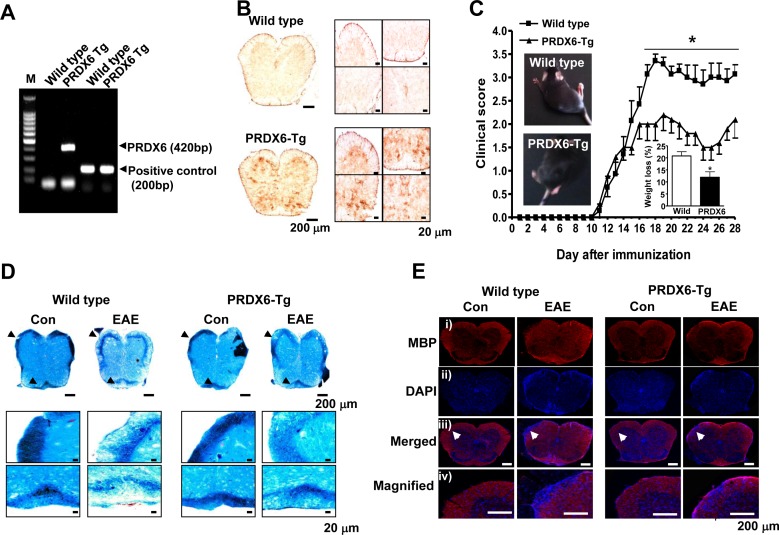
Transgenic (Tg) mice expressing PRDX6 are protected from EAE **A.** Ggenotyping was performed to analyze the existence of PRDX6 gene in transgenic mice, as described in Materials and Methods. **B.** The protein expression of PRDX6 was detected in the spinal cord of PRDX6 Tg mice and wild type mice using immunohistochemistry. **C.** Wild type and PRDX6 Tg mice were sensitized to MOG_35-55_, as described in Materials and Methods. Mice developed clinical signs of EAE starting at 11 days, characterized by an ascending flaccid paralysis, and were scored with widely used 5 point paradigm during 28 days once a day, as described in Materials and Methods. Inset, Representative picture of paralyzed mice and body weight loss were displayed in peak clinical scores. **D.**, **E.** Sections of spinal cord were stained with luxol fast blue solution after 28 days of EAE induction with MOG_35-55_ peptide **D.**. After sections of spinal cord were permeabilized, MBP (*i*; *red*) was immunostained with mouse anti-MBP antibody followed by Alexa-Fluor 568-conjugated secondary antibodies, and then sections were stained with DAPI (*ii*; *blue*). The *bottom* panels *(iii, iv)* show the merged images of the *first* (*i)* and *second* (*ii)* panels. *iv* panels show magnified image indicated with arrow in *iii* panels. Representative stainings from three independent experiments are shown. All values are presented as the mean ± SEM. *Significant difference between wild type mice and PRDX6 Tg mice sensitized with MOG_35-55_ peptide (*P < 0.05).

To investigate the role of PRDX6 in the disease progression of MS, PRDX6 transgenic (Tg) mice and non Tg mice (wild type mice) were used. The PRDX6 gene was confirmed by PCR of mouse tail genomic DNA in the PRDX6 Tg mice using allele-specific primers (Figure 2A). Immunohistochemisty revealed that PRDX6 was highly expressed in the spinal cord of PRDX6 Tg mice (Figure 2B). Upon EAE induction, we observed significant differences between wild type mice and PRDX6 Tg mice (Figure 2C). Clinical profiles revealed that both wild type mice and PRDX6 Tg mice exhibited neurological signs starting at the same time (11 days), but PRDX6 Tg mice was significantly less severe. The difference in clinical scores was evident at 16 days and sustained until the end point of the experiment (28 days). Together with clinical profiles, PRDX6 tg mice exhibited less weight loss than wild type mice (Figure 2C, *inset*). Additionally, we compared the highest clinical score and found that wild type mice displayed a mean peak clinical score of 3.357 ± 0.263, indicating severe paralysis, whereas PRDX6 Tg mice scored 2.250 ± 0.382, indicating hindlimb weakness (Figure 2C, *inset*).

To analyze the contribution of PRDX6 to demyelination from oligodendrocytes, we performed luxol fast blue (LFB) experiments to detect demyelination in the spinal cord of wild type mice and PRDX6 Tg mice. The LFB staining was remarkedly reduced on white matter of spinal cord in wild type mice compared to PRDX6 Tg mice (Figure 2D). Analysis of sections immunostained for myelin basic protein (MBP, myelin marker) also revealed that the decrease in myelin loss was observed in PRDX6 Tg mice compared to wild type mice (Figure 2E), indicating that PRDX6 prevented demyelination by EAE in the spinal cord.

### Up-regulation of PRDX6 in astrocytes of EAE and MS lesions, and blood-brain-barrier disruption

**Figure 3:**
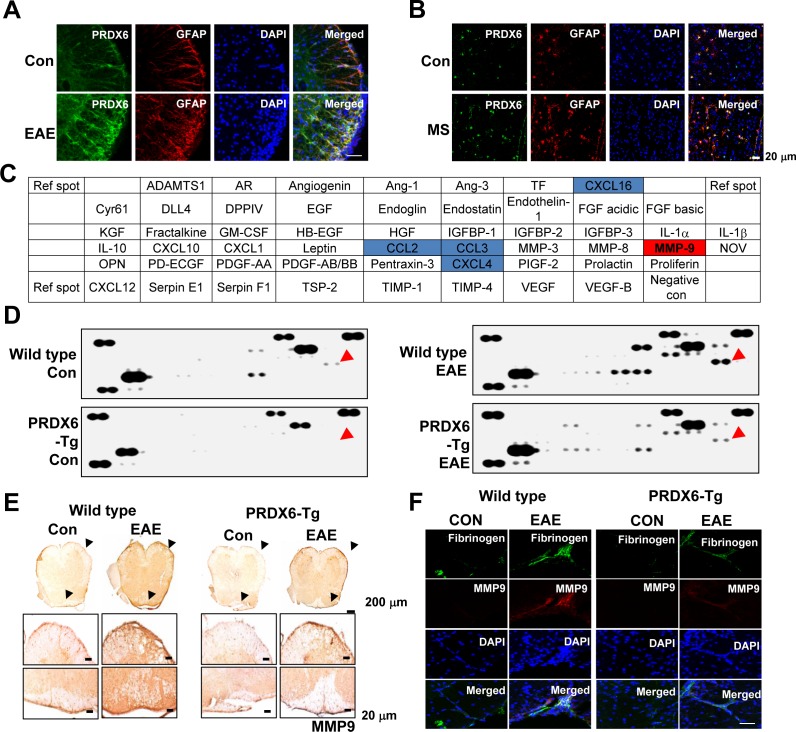
PRDX6 is increased in astrocytes and prevents blood-brain-barrier disruption **A.**, **B.** Double immunofluorescence for GFAP (red) and PRDX6 (green) is shown in the spinal cord of control (con) and EAE mice **A.** or human MS lesions **B.**. DAPI was used to stain for nuclei. The *last* panels show the merged images of the *first*, *second,* and *third* panels. **C.**, **D.** Mouse proteomic array. Nitrocellulose membranes contain 53 different protein antibodies printed in duplicate. Positive controls show the manufacturer's internal positive control samples on the membrane **C.**. Protein expression levels in the spinal cord of wild type and PRDX6 Tg mice were detected by Mouse proteomic array. Red arrow indicates MMP9. **D.**. **E.** The protein expression of MMP9 was detected in the spinal cord of PRDX6 Tg mice and wild type mice using immunohistochemistry. **F.** Immunofluorescence for MMP9 (*red*) and Fibrinogen (*green*) were immunostained with anti-MMP9 and anti-Fibrinogen antibodies in the spinal cord of PRDX6 Tg (*right*) and wild type mice (*red*), and then sections were stained with DAPI (*blue*). The *bottom panels* show the merged images of the *first, second*, and *third panels*. Representative stainings from three independent experiments are shown.

PRDX6 was specifically co-localzed with GFAP positive cells (astrocytes) in the spinal cord, but not co-localized in NeuN positive cells (neurons) and MBP positive cells (oligodendrocytes) (S2_Figure). PRDX6 was markedly increased in the spinal cord where EAE was induced with MOG_35-55_ peptide (Figure 3A). In human MS lesion, confocal imaging also demonstrated that PRDX6 was expressed and increased in GFAP positive cells (Figure 3B).

Next, we explored whether PRDX6 controlled a variety of growth factors, cytokines, and chemokines using proteomic array. MMP9 and chemokines such as CCL2, CCL3, CXCL4, and CXCL16 were changed between wild type mice and PRDX6 Tg mice sensitized with MOG_35-55_ peptides (and Figure 3C, 3D, and S3_Figure). Remarkably, MMP9 expression is increased in the spinal cord of wild type mice sensitized with MOG_35-55_ peptides, while the expression is decreased in the spinal cord of PRDX6 Tg mice sensitized with MOG_35-55_ peptides (Figure 3C and 3D). As shown in Figure 3E, the expression of MMP9 was also validated using IHC assay. MMP9 is mainly involved in BBB disruption. Consequently, fibrinogen (a marker of BBB disruption) showed strong staining in spinal cord of wild type mice sensitized with MOG_35-55_ peptides, whereas fibrinogen reduced by PRDX6 expression (Figure 3F). Gjb2 (gap junction protein, beta 2), Fn1 (fibronectin 1), and Ocel1 (occludin/ELL domain containing 1) involved in BBB disruption was also detected using a DNA microarray, and PRDX6 expression prevented the expression of the factors (Table 1 and S2_Dataset).

**Table 1:** Regulation of PRDX6, BBB, and chemokines by EAE induction in PRDX6-Tg mice

Probe ID	Ref Seq Accession	Gene Symbol	Entrez Gene ID	PRDX6 Tg/Wide type	Gene Name
A_55_P2176729	NM_007453	**Prdx6**	11758	**7.354452**	peroxiredoxin 6
A_55_P2176731	NM_007453	**Prdx6**	11758	**2.037113**	peroxiredoxin 6
A_52_P382886	NM_008125	**Gjb2**	14619	**−2.536138**	gap junction protein, beta 2
A_55_P2130178	NM_010233	**Fn1**	14268	**−2.248205**	fibronectin 1
A_55_P2180869	NM_029865	**Ocel1**	77090	**−2.314024**	occludin/ELL domain containing 1
A_52_P638459	NM_013653	**Ccl5**	20304	**−10.133732**	chemokine (C-C motif) ligand 5
A_52_P99888	NM_023158	**Cxcl16**	66102	**−2.677764**	chemokine (C-X-C motif) ligand 16
A_55_P2007964	NM_009987	**Cx3cr1**	13051	**−2.308**	chemokine (C-X3-C) receptor 1
A_55_P2054362	NM_009987	**Cx3cr1**	13051	**−2.525749**	chemokine (C-X3-C) receptor 1

### PRDX6 controls chemokines, immune cell infiltration, and neuroinflammation

**Figure 4:**
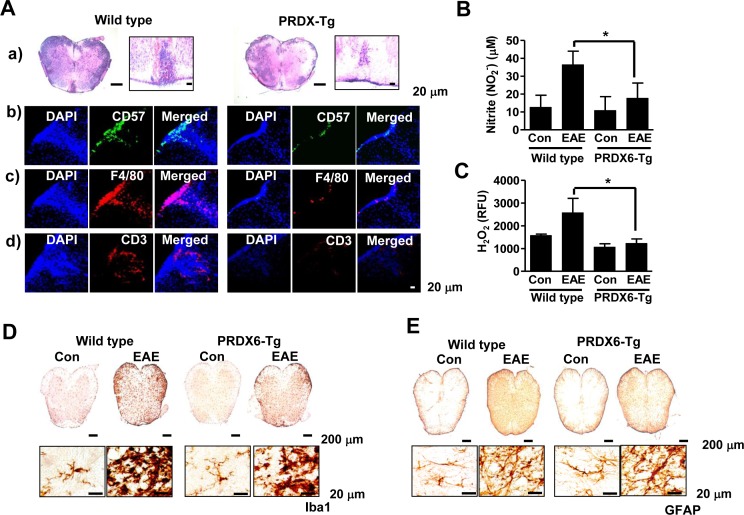
PRDX6 attenuates immune cell infiltration and neuroinflammation in EAE **A.**, **B.** Spinal cord sections were incubated with specific antibodies against GFAP **A.** and Iba1 **B.** proteins and then were detected by immunohistochemistry. **C.** Images show hematoxylin and eosin (H&E) staining of the spinal cord of PRDX6 Tg (right) and wild type mice (left) at 28 days after immunization with MOG35-55 peptide **A.**. CD57 (*b, green*), F4/80 (*c, red*), and CD3 (*d, red*) was immunostained in PRDX6 Tg mice (right) and wild type mice (left) spinal cord, and then sections were stained with DAPI (*blue*). The *last* panels show the merged images of the *first* and *second* panels. **D.**, **E.** Nitric oxide (NO) levels **D.** and reactive oxygen species (ROS) levels **E.** were determined using NO detection kit and ROS detection kit, respectively. All values are presented as the mean ± SEM. *Significant difference between wild type mice mice and PRDX6 Tg mice that were sensitized with MOG_35-55_ peptide (*P < 0.05). Representative data from three independent experiments are shown.

We found that PRDX6 prevented BBB disruption as well as the expression of chemokines including CCL2, CCL3, CCL5, CX3CR1, CXCL4, and CXCL16 (Figure 3, S3_Figure, Table 1, and S2_Dataset). Therefore, we investigated immune cell infiltration in the EAE pathophysiology. Sections of the spinal cord from mice in each group were immunostained for NK cells (CD57), macrophages (F4/80), and T lymphocytes (CD3). The results showed that immune cell infiltration was almost halved in the spinal cord of PRDX6 Tg mice compared with wild type mice. Characterization of inflammatory infiltrates indicated reduction in T lymphocytes, NK cells, and macrophages in PRDX6 Tg mice (Figure 4A). DNA microarray also showed that EAE-induced PRDX6 Tg mice suppressed immune response. (S2_Table and S2_Dataset). It was also found that NO and ROS generation were induced following sensitization with MOG_35-55_, but were remarkably decreased by PRDX6 in the spinal cord (Figure 4B and 4C). It is known that the activation of microglia results in neuroinflammation. Sections of the spinal cord from mice in each group were subjected to immunohistochemistry for markers of microglia (Iba1). As a result, Iba1 reactive cell number were markedly increased in the spinal cord of wild type mice sensitized with MOG_35-55_ peptides, as well as the morphological changes indicated the activation of microglia. However, the activation of microglia was attenuated in the spinal cord of PRDX6 Tg mice sensitized with MOG_35-55_ peptides (Figure 4D). Immunohistochemistry for GFAP showed GFAP reactive cell numbers and morphological changes were markedly increased in the spinal cord of wild type mice sensitized with MOG_35-55_ peptides (Figure 4E).

## DISCUSSION

In the present study, we originally demonstrated that PRDX6 prevents from intensified clinical deficits, more extensive demyelination and neurodegeneration, heightened CNS inflammation and BBB disruption.

Reactive astrocytes are one of the earliest histopathological signs in MS lesions [12]. Astrocytes increased inducible nitric oxide synthase expression, and produce highly damaging reactive oxygen and nitrogen species[13-14]. In the present study, generation of ROS and NO was induced following EAE, while PRDX6 decreased the generation of ROS and NO in the spinal cord. We also found that PRDX6 was significantly increased in reactive astrocytes under EAE. High levels of NO and superoxide have all been reported in patients with MS [15]. Their excessive amounts are noxious to cause cellular damage and cell metabolism such as apoptosis [16]. Although Holley et al. showed that PRDX5 is prominently expressed in astrocytes of acute, subacute and chronic lesions of multiple sclerosis patients [17], our data demonstrated that PRDX5 expression in EAE is consistent with normal condition. Thus, our data suggest that the upregulation of PRDX6 in reactive astrocytes inhibits EAE via reduction of ROS and NO.

BBB disruption and extravasation of immune cells into the brain parenchyma are the earliest steps in the pathogenesis of MS [18–19] and MMP9 is the most important in promoting BBB disruption among the various MMPs associated with CNS inflammation [20]. In the present study, we detected peripheral immune cell infiltration into the spinal cord, the activation of microglia, and MMP9 and the blood protein fibrinogen leakage which are involved in BBB disruption and immune cell infiltration. The inhibition of MMP9 improved the clinical condition by blocking the BBB injury [21], and suppressed inflammatory reactions in activated microglia [22]. Konnecke et al demonstrated that microglia is main sources of MMP9 in neuroinflammation [23]. Similarly, we also demonstrated that upregulation of PRDX6 decreased MMP9 expression and fibrinogen leakage with reduction of immune cell infiltration and microglia activation during EAE. Therefore, the upregulation of PRDX6 in astrocytes inhibits microglial activation, MMP9 expression, and the prevent BBB breakdown and immune cell infiltration, leading to protection from the damage of the spinal cord by EAE.

Several studies have indicated that astrocytes increase secretion of anti-inflammatory cytokines and scavengers of ROS, as well as the suppression of microglial activation is caused by astrocyte induced anti-inflammatory function [3, 24–26]. These studies also support our idea for neuroprotective effects of astrocytic PRDX6 as a critical factor in EAE mouse model and MS lesion. In conclusion, our findings strongly support that upregulation of astrocytic PRDX6 has an important role in orchestrating myelin destruction via microglial activation, BBB breakdown, and immune cell infiltration, resulting in demyelination and axonal loss in MS, as well as our data provide the concept that PRDX6 gives rise to great potential in prevention and treatment of MS.

## MATERIALS AND METHODS

### Ethics statement

All animal experiments were approved and carried out according to the Guide for the Care and Use of Animals [Chungbuk National University Animal Care Committee, Korea (CBNUA-045-0902-01)].

### Genomic DNA and PCR

The C57BL/6 J-Tg (PRDX6) mice were purchased from The Jackson Laboratory (Bar Harbor, Maine). PRDX6 insertion was confirmed by the amplification of genomic DNA isolated from the transgenic mice tails using Super Taq PLUS Pre-mix (RexGene BioTech, Korea) and the following specific primer set: PRDX6 sense, 5-CAA GGT AGT GTC AGT GTG G-3; and PRDX6 antisense, 5-GTA ATA CGA CTC ACT ATA GGG CG-3 (420 bp); positive control sense, 5- CAA ATG TTG CTT GTC TGG TG-3; and positive control antisense, 5-GTC AGT CGA GTG CAC AGT TT-3 (200 bp). Genomic DNA samples were extracted from transgenic mice tails using G-spin Total DNA Extraction Kit (iNtRON Biotech,nology, Inc., South Korea) and PCR analysis was performed for detection of insertion of PRDX6 in the genome.

### EAE induction

Mice (13 weeks old, *n* = 10) were immunized with MOG_35-55_/CFA Emulsion PTX, Hooke kits (#EK-2110, Hooke laboratories, Lawrence, MA) according to the manufacturer's instructions. Briefly, 0.1 ml MOG35-55/CFA emulsion was injected subcutaneously into both flanks of each mouse (0.2 ml/animal). Then, the mice received intraperitoneal injections of pertussis toxin (PTX) (0.1 ml/animal/day, i.p.) on the same day and 24 hours later. The day after the last injection of MOG was considered day 1. Clinical signs were scored as follows: 0, no clinical sign; 0.5, partial tail paralysis; 1.0, complete tail paralysis; 1.5, complete tail paralysis and discrete hind limb weakness; 2.0, complete tail paralysis and strong hind limb weakness; 2.5, unilateral hind limb paralysis; 3, complete hind limb paralysis; 3.5, hind limb paralysis and forelimb weakness; 4.0, complete paralysis (tetraplegia), and 5.0, moribund or dead.

### Reverse transcriptase-polymerase chain reaction (RT-PCR) and quantitative real-time PCR

The total RNA of cells was extracted with Trizol reagent (Life Technologies, Gaithersburg, MD) according to the manufacturer's instructions. The generated cDNAs with AccuPower RT PreMix (iNtRON Biotechnology, Gyeonggi-do, South Korea) were amplified with AccuPower PCR PreMix (Bioneer Corporation, Daejeon, South Korea). The sequences of primers are listed in S3_Table. For mRNA quantification, total RNA was extracted using the RNAqueous kit and the cDNA was synthesized using High Capacity RNA-to-cDNA kit (Applied Biosystems, Foster City, CA) according to the manufacturer's protocol. Quantitative real-time PCR was performed using a 7500 Real-Time PCR System (Applied Biosystems).

### Gene expression profiles

Total RNAs of spinal cord tissues were extracted with RNeasy (QIAGEN Inc., Valencia, CA) according to the manufacturer's instructions. The gene expression profiles were analyzed with Macrogen Inc. (Seoul, South Korea) using 8x60K high density SurePrint G3 gene expression mouse Agilent microarray. (Agilent, Santa Clara, CA, USA).

### Immnohistochemistry (IHC) and immunofluorescence (IF)

The spinal cord was cut into 18 μm coronal sections with a freezing microtome (Thermo Scientific, Germany) and stored in cryoprotectant solution at −20 °C. The sections or MS patient sections (Gentex, Zeeland, MI) were subject to immunostainings. Briefly, Endogenous peroxidase activity was quenched, and the sections were blocked for 30 min with 3% normal horse/goat serum diluted in PBS. These sections were incubated with specific primary antibody for overnight. After washing in PBS, the sections were incubated in biotinylated goat anti-mouse/rabbit IgG antibody (1:1000 dilution, Vector Laboratories, Burlingame, CA, USA) for 1 h at room temperature. The sections were subsequently washed and incubated with avidin-conjugated peroxidase complex (ABC kit, 1:200 dilution, Vector Laboratories, Burlingame, CA, USA) for 30 min followed by PBS washing. The peroxidase reaction was performed in PBS using 3, 3′-diaminobenzidine tetrahydrochloride (DAB, 0.02%). Sections were dehydrated in ethanol, cleared in xylene, and mounted with Permount (Fisher Scientific), and evaluated on a light microscopy (Olympus, Tokyo, Japan).

For Immunofluorescence, sections were incubated with specific primary antibody for overnight at 4 °C, and then labeled with an anti-rabbit secondary antibody conjugated with Alexa-Fluor 488 (1:500 dilution, Invitrogen) or anti-mouse secondary antibody conjugated with Alexa-Fluor 568 (1:500 dilution, Invitrogen) at the darkness for 2 h at room temperature. Final images were acquired using a confocal laser scanning microscope (TCS SP2, Leica Microsystems AG, Werzlar, Germany).

### Hematoxylin-eosin (H&E) and Luxol fast blue staining

Sections were for stained with H&E for routine histological analysis of inflammatory infiltration and with Luxol fast blue stain kits (IHC WORLD, LLC., Woodstock, MD) for evaluation of demyelination.

### Proteome profilerTM antibody arrays

The spinal cord tissues were excised and homogenized in PBS with protease inhibitor cocktail (Sigma-Aldrich, USA) and Triton X-100 (final concentration 1%). 300 μg of proteins collected from three samples per group was used for Proteome Profiler^TM^ Antibody Arrays according to the protocol provided by supplier (#ARY015, R&D Systems, USA).

### ROS determination

Intracellular generation of ROS was monitored using OxiSelect™ Hydrogen Peroxide / Peroxidase Assay Kit (Cell Biolabs, Inc., San Diego, CA). To investigate ROS generation, the spinal cord tissues were homogenized in 1X Assay Buffer. The lysates were assayed according to manufacturer's procedure.

### Nitric oxide (NO) determination

100 μl of the spinal cord tissues was mixed with an equal volume of Griess reagent [0.1% N-(1-naphthyl)- ethylenediamine, 1% sulfanilamide in 5% phosphoric acid] (Sigma) and incubated at room temperature for 10 min. The absorbance at 540 nm was measured in an automated microplate reader (Molecular Decices), and a series of known concentrations of sodium nitrite was used as a standard.

### Statistical analysis

The data were analyzed using the GraphPad Prism version 4 program (GraphPad Software, Inc., San Diego, CA). Data are presented as mean ± SEM. Statistical significance was performed on the data using one-way analysis of variance (ANOVA) or unpaired Student's *t* test. A value of *P* < 0.05 was considered to be statistically significant.

## SUPPLEMENTARY MATERIAL FIGURES AND TABLES






