# Supplementary material for: PRDX6 controls multiple sclerosis by suppressing inflammation and blood brain barrier disruption
Source: Oncotarget. 2015 Aug 17;6(25):20875–84. doi: 10.18632/oncotarget.5205 (PMC4673236; doi:10.18632/oncotarget.5205)
Supplement: Supplementary file 1 [file oncotarget-06-20875-s001.pdf]

PRDX6 controls multiple sclerosis by suppressing inflammation and blood brain barrier disruption

Supplementary Material

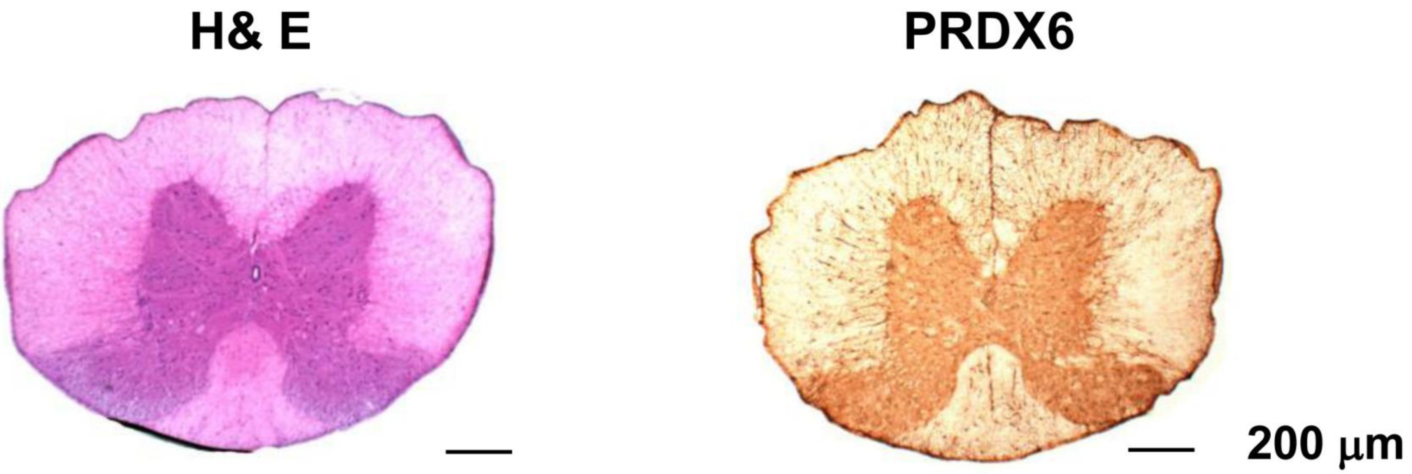

S_Figure 1. Expression of PRDX6 protein in mouse spinal cord. Images show hematoxylin and eosin (H&E) staining of mouse spinal cord (*left*). The spinal cord section was incubated with specific antibodies against PRDX6 protein and then were detected by immunohistochemistry (*right*).

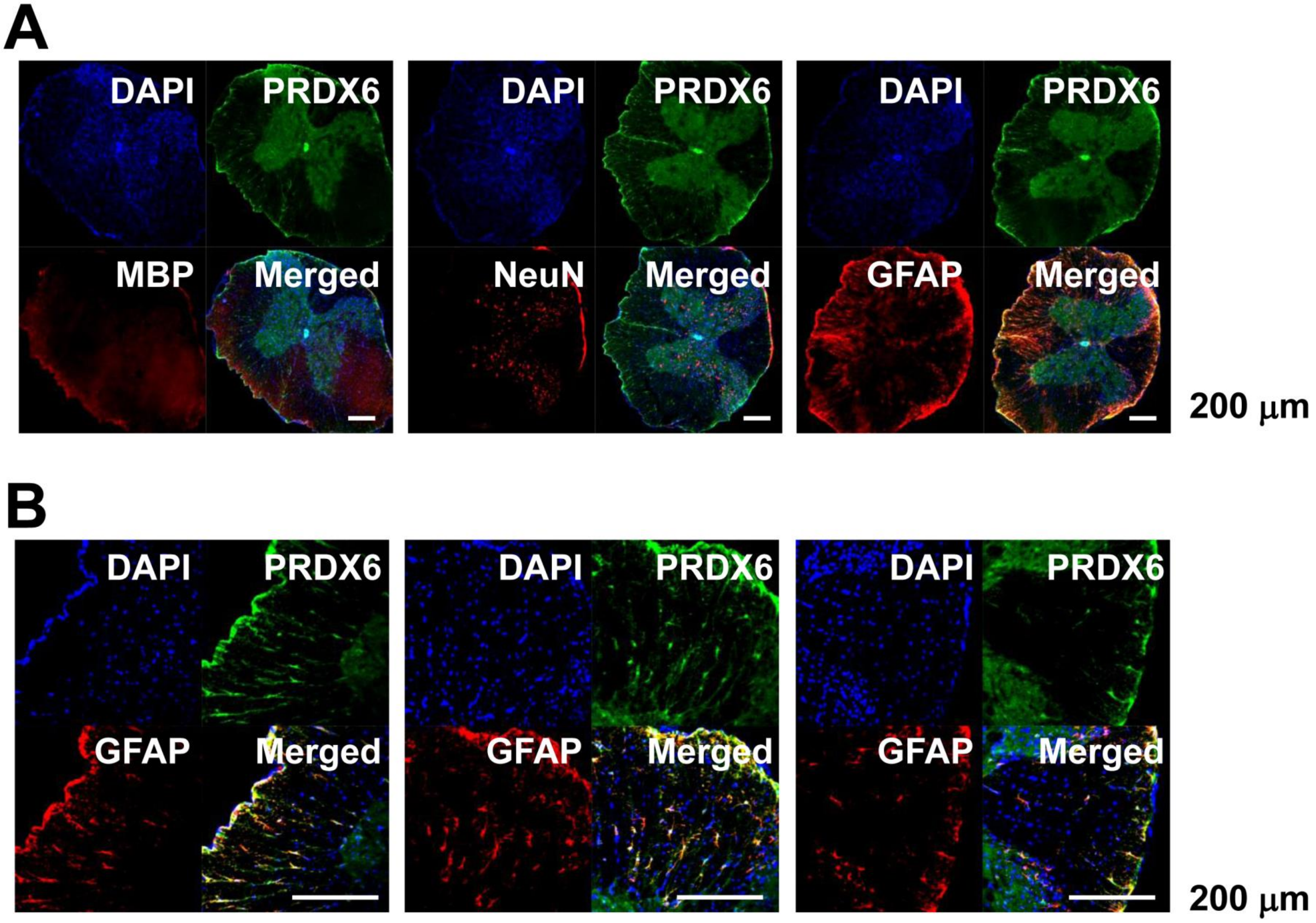

S_Figure 2. Specific epression of PRDX6 on astrocytes in mouse spinal cord. (A) Immunofluorescence for PRDX6 (green) and MBP (red, oligodenrocyte marker), NeuN (red, neuron marker) or GFAP (red, astrocytes marker) was immunostained with rabbit anti-PRDX6 and mouse anti-MBP, -NeuN, or -GFAP antibodies in the mouse spinal cord, and then, sections were stained with DAPI (blue). (B) Magnified images show region containing dorsal and ventral column from immunofluorescence for PRDX6 and GFAP of *A*.

Ref spot		ADAMTS1	AR	Angiogenin	Ang-1	Ang-3	TF	CXCL16		Ref spot
	Cyr61	DLL4	DPPIV	EGF	Endoglin	Endostatin	Endothelin-1	FGF acidic	FGF basic	
	KGF	Fractalkine	GM-CSF	HB-EGF	HGF	IGFBP-1	IGFBP-2	IGFBP-3	IL-1α	IL-1β
	IL-10	CXCL10	CXCL1	Leptin	CCL2	CCL3	MMP-3	MMP-8	MMP-9	NOV
	OPN	PD-ECGF	PDGF-AA	PDGF-AB/BB	Pentraxin-3	CXCL4	PIGF-2	Prolactin	Proliferin	
Ref spot	CXCL12	Serpin E1	Serpin F1	TSP-2	TIMP-1	TIMP-4	VEGF	VEGF-B	Negative con	

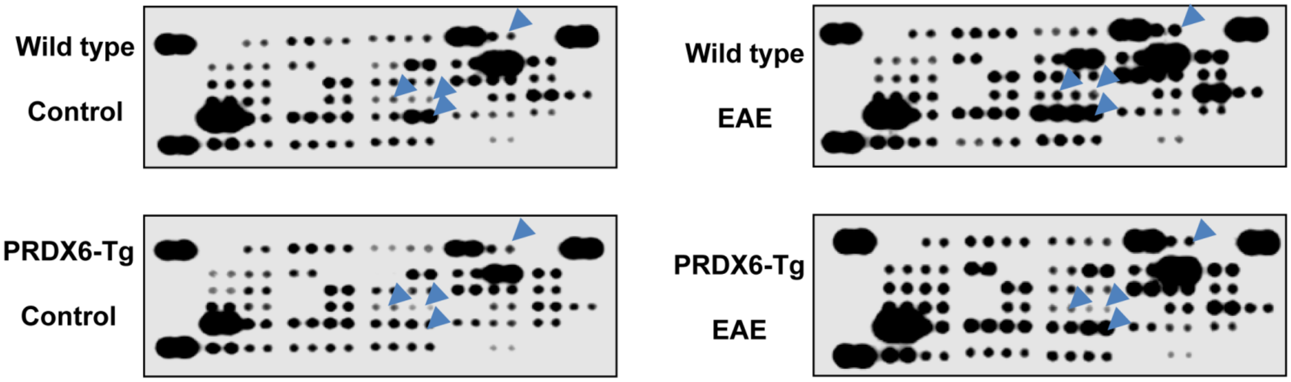

S3_Figure. The long exposure band and original membrane of Mouse proteomic array in Figure 3C, D. PRDX6 attenuates the expression of chemokines. Blue arrow indicates CCL2, CCL3, CXCL16, and CXCL4.

S1_Table. Upregulation of PRDX family in the EAE mouse model.

**Probe ID**	**Ref Seq Accession**	**Gene Symbol**	**Entrez Gene ID**	**PRDX6 Tg/ Non Tg**	**Gene Name**
A_55_P2147427	NM_011034	Prdx 1	18477	**2.527413**	peroxiredoxin 1
A_55_P2176729	NM_007453	Prdx 6	11758	**3.61913**	peroxiredoxin 6
A_55_P2176731	NM_007453	Prdx 6	11758	**3.245044**	peroxiredoxin 6
A_55_P2103561	NM_007453	Prdx 6	11758	**2.489484**	peroxiredoxin 6

S2_Table. Differentiation between non-Tg EAE and PRDX6 EAE in immune system

Probe ID	Ref Seq Accession	Gene Symbol	Entrez Gene ID	PRDX6 Tg/ Non Tg	Gene Name
A_52_P343306	NM_010378	H2-Aa	14960	-7.045759	histocompatibility 2, class II antigen A, alpha
A_55_P2062246	NM_001145164	Tgtp2	1E+08	-5.215047	T cell specific GTPase 2
A_55_P1981494		H2-Bl	14963	-4.094391	histocompatibility 2, blastocyst
A_55_P2049647	NM_001001892	H2-K1	14972	-4.226228	histocompatibility 2, K1, K region
A_55_P1962747	NM_207105	H2-Ab1	14961	-4.701439	histocompatibility 2, class II antigen A, beta 1
A_55_P2146560	NM_207105	H2-Ab1	14961	-4.576559	histocompatibility 2, class II antigen A, beta 1
A_55_P2156731	NM_010382	H2-Eb1	14969	-4.79502	histocompatibility 2, class II antigen E beta
A_55_P1978465	NR_051981	H2-Q5	15016	-3.901872	histocompatibility 2, Q region locus 5
A_51_P266248		Ighv14-2	668421	-3.746982	immunoglobulin heavy variable 14-2
A_51_P198434	NM_001001892	H2-K1	14972	-4.226228	histocompatibility 2, K1, K region
A_55_P2049647	NM_001001892	H2-K1	14972	-3.609212	histocompatibility 2, K1, K region
A_51_P237754	NM_010398	H2-T23	15040	-3.333856	histocompatibility 2, T region locus 23
A_51_P351860	NM_009777	C1qb	12260	-3.308733	complement component 1, q subcomponent, beta polypeptide
A_55_P1978506	NM_023124	H2-Q8	15019	-3.164477	histocompatibility 2, Q region locus 8
A_51_P497171	NM_008534	Ly9	17085	-3.107275	lymphocyte antigen 9
A_51_P219789	NM_010392	H2-Q2	15013	-3.072134	histocompatibility 2, Q region locus 2
A_51_P390538	NM_010821	Mpeg1	17476	-3.048441	macrophage expressed gene 1
A_55_P1978424	NM_007536	Bcl2a1d	12047	-2.982013	B cell leukemia/lymphoma 2 related protein A1d
A_51_P219789	NM_010392	H2-Q2	15013	-2.892238	histocompatibility 2, Q region locus 2
A_51_P502456	NR_004446	H2-K2	630499	-2.832626	histocompatibility 2, K region locus 2
A_51_P377452	NM_008677	Ncf4	17972	-2.836214	neutrophil cytosolic factor 4
A_65_P19395	NM_010380	H2-D1	14964	-2.797097	histocompatibility 2, D region locus 1
A_51_P102789	NM_007574	C1qc	12262	-2.781987	complement component 1, q subcomponent, C chain
A_51_P110301	NM_009778	C3	12266	-2.765408	complement component 3
A_51_P502456	NR_004446	H2-K2	630499	-2.737348	histocompatibility 2, K region locus 2
A_51_P469968	NM_013819	H2-M3	14991	-2.701597	histocompatibility 2, M region locus 3
A_55_P1978511	NM_010394	H2-Q7	15018	-2.70247	histocompatibility 2, Q region locus 7
A_51_P278868	NM_010387	H2-DMb1	14999	-2.623058	histocompatibility 2, class II, locus Mb1
A_55_P2015363	NM_023143	C1ra	50909	-2.190626	complement component 1, r subcomponent A

Table 4. Reverse transcriptase-polymerase chain reaction (RT-PCR) primers and conditions.

Genes	Primer Sequence (5'-3')	Annealing Temp (℃)	Cycle Number
Prdx1	F : 5'- TTTACCTGCCTGTTGGATACC -3'<br>R : 5'- GGAGAGACAGCTCAATGGTTAG -3'	56	30
Prdx2	F: 5'- TGCTTGCTGACGTGACTAAA -3'<br>R: 5'- TTGACTGTGATCTGGCGAAG -3'	56	30
Prdx3	F: 5'- CTAGGGACTTCTTGATGGCTAAC -3'<br>R:5'- GGCAGGCTAAGGGAAAGAAT -3'	56	30
Prdx4	F: 5'- GACACTGCGTTTGGTTCAAG -3'<br>R: 5'- CCAGCTGGATCTGGGATTATT -3'	56	30
Prdx5	F: 5'- GGGAAGGCGACAGACTTATTATT -3'<br>R: 5'- CCTTCACTATGCCGTTGTCTATC -3'	56	30
Prdx6	F : 5'- GCTTCCTTCTTGCTGGGAATAG -3'<br>R : 5'- GGAGAACAGGAACCAAGGAATTAG -3'	56	30
β-actin	F : 5'- AATGTGGCTGAGGACTTTG -3'<br>R : 5'- GGGACTTCCTGTAACCACTTATT -3'	58	26
